# The influence of light path length on the color of synthetic ruby

**DOI:** 10.1038/s41598-022-08811-y

**Published:** 2022-04-08

**Authors:** Bin Yuan, Ying Guo, Ziyuan Liu

**Affiliations:** Department of Gemmology, China University of Geosciences, Beijing, 100083 China

**Keywords:** Materials science, Optics and photonics

## Abstract

The corrected ultraviolet–visible light spectrum was used to calculate the color of synthetic rubies with different light path lengths, and the influence of light path length and standard light source on the color of synthetic ruby was studied. The results show that the difference in colour between the o light direction and the e light direction of the synthetic ruby decreases as the length of the light path increases. At the same time, as the length of the light path increases, the lightness L* decreases, and the hue angle h° increases. The chroma C* first increases as the length of the light path increases, and then begins to decrease under the influence of the continuous decrease in lightness. The color difference ΔE*_ab_ reaches the maximum when the light path length is around 10 mm, and the standard light source has the greatest influence on the color difference ΔE*_ab_. As the length of the light path continues to increase, the influence of the standard light source on the color difference ΔE*_ab_ decreases. In the ultraviolet–visible light spectrum, the strong absorption band of Cr^3+^ at 545 nm is the main cause of the color of the ruby. The larger the area of the band at 545 nm, the lower the lightness and the higher the hue angle, which means the ruby colour is redder.

## Introduction

Ruby is a kind of corundum with a beautiful bright red color. It is widely distributed all over the world, such as Myanmar, Thailand, Sri Lanka, Tanzania, etc. When Al in corundum is replaced by various elements such as Cr, Fe, Ti, and V, the corundum will show various colors. The color of ruby is related to Cr^3+^. And as the replacement of Al^3+^ by Cr^3+^ increases, the color will change from light pink to red^1,2^ flame-fusion synthetic ruby is the most common type of synthetic ruby. Compared with natural ruby, it has a pure texture and is more suitable for chromaticity research.

There are many ways to evaluate the color of gems. The most common is the Cape series diamond color grading. The grader compares the diamond to be graded with a standard colorimetric stone to determine the color grade of the diamond^3–5^. However, in addition to diamonds, it is difficult to determine the color grade of other gemstones in this way, and because it is artificially graded, there may be large errors. The CIE standard colorimetric system can effectively solve this problem, of which the CIE1931-XYZ system and the CIE1976L*a*b uniform color space are the most widely used. Stockton^6^ first used the Gem ColorMaster instrument to quantify the color of peridot in the CIE1931 color space. But the CIE1931-XYZ system is a non-uniform color space and cannot describe the color of gems well. The CIE1976L*a*b uniform color space is modified based on the CIE1964 uniform color space, and it is widely used in gem color evaluation^7–12^.

The main instrument currently used to measure the color of gemstones is a spectrophotometer. The portable spectrophotometer is used to measure the color of perdiot^13–15^, amethyst^16^, turquoise^17^, rubellite^18^, and green chrysoprase^19,20^. Color i5 and GemDialogue color cards are used to quantitatively describe the color of jadeite^21^. In addition, computer vision systems can also be used to measure the color of jadeite^22^. Because the portable spectrophotometer is a closed system, it cannot effectively reflect the pleochroism of some gemstones. The use of ultraviolet–visible spectrophotometer can effectively solve this problem^23^. Compared with the portable spectrophotometer, the UV–Vis spectrophotometer measures color more objectively and accurately. The UV–Vis spectrophotometer calculates the colors of leaves^24^ and flowers^25^, garnets^26,27^, synthetic alexandrite^28^, dyed purple opal^29^, etc. by using CIE1931RGB and CIEXYZ color matching functions.

The human retina has three kinds of color photoreceptor cells, namely cone cells, that are sensitive to red, green, and blue light. S cones detect short wavelength (blue), M cones detects medium wavelength (green), L cones detect long-wavelength (red). When exposed to radiation, the spectral stimulus energy is absorbed by photoreceptors of the three cones. The cone cells produce different degrees of neurophysiological reactions. The International Commission on Illumination (CIE) has established a series of color matching functions through visual experiments. As a proxy for the cone response function, the CIE color-matching functions are used to express the linear combination of the average visual response^30^. The matching function can be used to calculate the energy of light that enters the human eye and produces the color perception. Sun^28^ discussed the influence of different path lengths on the color of synthetic Cr-bearing chrysoberyl by calculating the color matching function. In addition, pleochroism is also a factor that needs to be considered when studying the color of gems^31^.

**Figure 1:**
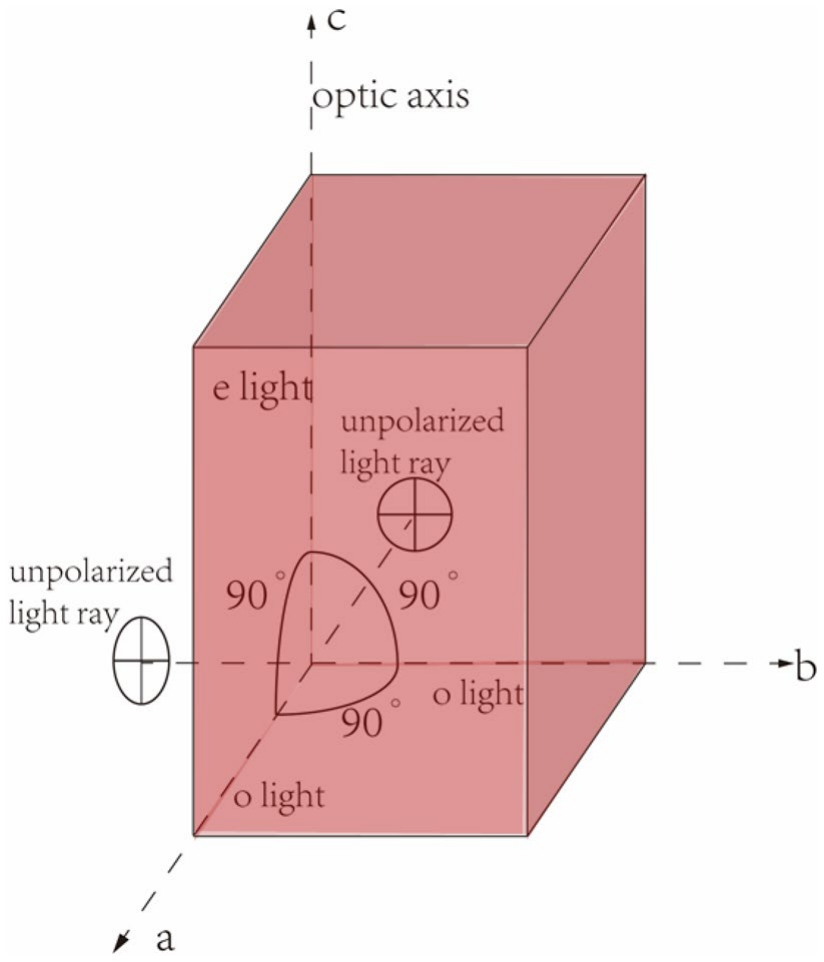
Schematic diagram of the sample. Three mutually perpendicular crystal axes a, b, and c. The unpolarized light parallel to the a-axis and the b-axis is split into o-light and e-light when passing through the crystal, and the unpolarized light parallel to the c-axis passes through the crystal along the optical axis.

Two fully polished synthetic ruby cuboids R and Ru which have been oriented by an X-ray crystal orientation instrument are selected. Size is 5.89 mm × 5.97 mm × $9.29 \; \mathrm{mm}$ and 5.46 mm $\times \, 5.41 \; \mathrm{mm}\times 8.9 \; \mathrm{mm}$. The sample is shown in Fig. 1. The height is the c-axis, and the surface is perpendicular to the optical axis. The length and width of the gemstone are selected as the a-axis and b-axis. The a, b, and c axes of the two samples are numbered respectively R_oa_, R_ob_, R_ec_, Ru_oa_, Ru_ob_, Ru_ec._ This paper mainly uses the color matching function to quantitatively characterize the color, and studies the influence of the light path length and the light source on the color of the ruby.

## Results and discussion

### UV–Vis spectral analysis

**Figure 2:**
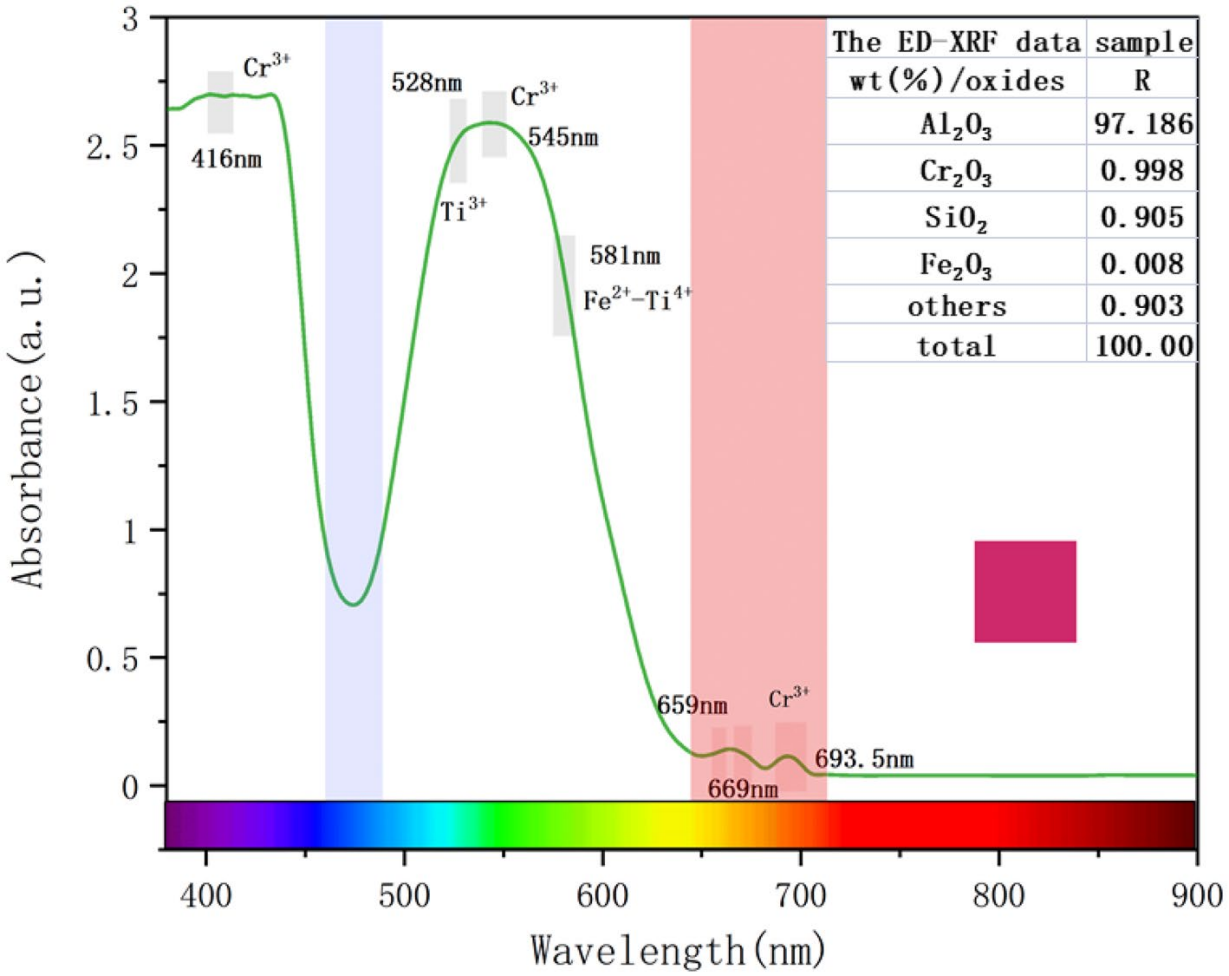
Ultraviolet–visible light spectrum of synthetic ruby R_oa_. Two transmission windows appear at 652–700 and 465–480 nm, which transmit red light and a small amount of blue-violet light. The ED-XRF data of sample R is shown in the upper right corner.

The UV–Vis spectrum of synthetic ruby is shown in Fig. 2. The top right-hand corner of Fig. 2 shows the ED-XRF data for synthetic ruby. ED-XRF is a semi-quantitative chemical composition test that can be used to quickly detect the content of most elements in synthetic ruby. It can be seen that there are obvious absorption peaks at 693.50 nm, 669 nm, and 659 nm, as well as broad absorption bands centered at 545 nm and 416 nm. The absorption peak at 693.50 nm is the fluorescence emission peak caused by Cr^3+^, which is caused by ^2^E → ^4^A_2_ of Cr^3+^, which is the reason for the strong fluorescence of synthetic ruby. The two weak absorption peaks at 669 nm and 659 nm are the ^4^A_2_ → ^2^T_1_ transition caused by Cr^3+^. The absorption peak near 581 nm is related to the charge transfer of Fe^2+^-Ti^4+^. The absorption peak at 528 nm is related to the ^2^D splitting of the Ti^3+^ spectrum item^32,33^. The absorption peaks at 545 nm and 416 nm are the ^4^A_2_ → ^4^T_2_ transition caused by Cr^3+^, which absorb yellow-green light, allowing red light and a small amount of blue-violet light to pass through, forming the color of synthetic ruby. As can be seen from the ED-XRF data in Fig. 2, the main chemical composition of the synthetic ruby is Al_2_O_3_, which accounts for 97.186%. The colour-causing elements Cr_2_O_3_ and Fe_2_O_3_ account for 0.998% and 0.008% respectively. This coincides with the absorption peaks in the UV–Vis spectrum of Fig. 2.

### Correcting the UV–Vis spectra

When light passes through the sample, energy is lost in three ways. A is the total absorbance of the sample measured directly from the spectrophotometer, including A_c_ (absorbance contribution of the absorber), A_rl_ (absorption caused by light reflection at the boundary), A_isl_ (absorption caused by scattering of internal inclusions)^26,28^.

1 $$\mathrm{A}={A}_{c}+{A}_{rl}+{A}_{isl}$$

There are many different methods to correct the baseline. For example, Sun^26^ subtracted the absorption spectrum at 800 nm to obtain a corrected spectrum. In this study, the Sellmeier equation is used to eliminate Arl and to correct the baseline of the ultraviolet–visible spectrum. At the same time because the sample is synthetic ruby with pure texture and no inclusions, Aisl does not affect the UV–Vis spectrum. Thus, the corrected UV–Vis spectrum is obtained.

The absorption (A_rl_) of light reflected at the boundary is related to the refractive index (n) of the sample. The Sellmeier equation is an empirical formula that describes the refractive index and wavelength in a specific transparent medium and is used to determine the dispersion of light in the medium. Different materials have different Sellmeier coefficients. According to the research of Malitson^34^, we get the following Sellmeier formula:

2 $${\mathrm{n}}^{2}\left(\uplambda \right)=1+\frac{{\mathrm{B}}_{1}{\uplambda }^{2}}{{\uplambda }^{2}-{\mathrm{C}}_{1}}+\frac{{\mathrm{B}}_{2}{\uplambda }^{2}}{{\uplambda }^{2}-{\mathrm{C}}_{2}}+\frac{{\mathrm{B}}_{3}{\uplambda }^{2}}{{\uplambda }^{2}-{\mathrm{C}}_{3}}$$

where n is the refractive index, $\uplambda$ is the wavelength, and B_1,2,3_ and C_1,2,3_ are different Sellmeier coefficients. For corundum, there are the following Sellmeier equations in the direction of the corundum o light and the direction of the e light respectively:

3 $${\mathrm{n}}^{2}\left(\uplambda \right)=1+\frac{1.4313493{\uplambda }^{2}}{{\uplambda }^{2}-{0.0726631}^{2}}+\frac{0.65054713{\uplambda }^{2}}{{\uplambda }^{2}-{0.1193242}^{2}}+\frac{5.3414021{\uplambda }^{2}}{{\uplambda }^{2}-{18.028251}^{2}}$$

4 $${\mathrm{n}}^{2}\left(\uplambda \right)=1+\frac{1.5039759{\uplambda }^{2}}{{\uplambda }^{2}-{0.0740298}^{2}}+\frac{0.55069141{\uplambda }^{2}}{{\uplambda }^{2}-{0.1216529}^{2}}+\frac{6.5927379{\uplambda }^{2}}{{\uplambda }^{2}-{20.072248}^{2}}$$

It is assumed that in the ideal state when unpolarized light is incident perpendicularly to the surface of the gemstone, a certain length of optical path will not be generated inside. In this case, the light absorption inside the gem can be ignored. Therefore, the transmittance through the surface of the gemstone can be expressed as:

5 $$\mathrm{T}=1-\mathrm{R}$$

According to Lambert Beer’s law, transmittance can be converted to absorbance:

6 $$\mathrm{A}=\mathrm{lg}\frac{1}{\mathrm{T}}=\mathrm{kbc}$$

where A is the absorbance, T is the transmittance, k is the molar absorbance coefficient, c is the concentration of the absorbing substance, and b is the optical path of the light.

7 $$\mathrm{T}={10}^{-\mathrm{A}}=1-\mathrm{R}$$

8 $$\mathrm{A}=-\mathrm{lgT}=-\mathrm{lg}\left(1-\mathrm{R}\right)$$

9 $$\mathrm{R}={ \left(\frac{{\mathrm{n}}_{0}-{\mathrm{n}}_{1}}{{\mathrm{n}}_{0}+{\mathrm{n}}_{1}} \right)}^{2}$$

10 $${A}_{rl}=2A=-2lg\left[1-{ \left(\frac{{\mathrm{n}}_{0}-{\mathrm{n}}_{1}}{{\mathrm{n}}_{0}+{\mathrm{n}}_{1}} \right)}^{2}\right]$$

R is the reflectivity, n_0_ = 1 is the refractive index of light in the air, n_1_ is the refractive index of light in the corundum, T is the transmittance, and A is the absorbance produced by single boundary reflection. A_rl_ is the absorbance produced by the reflection of the two boundaries.

**Figure 3:**
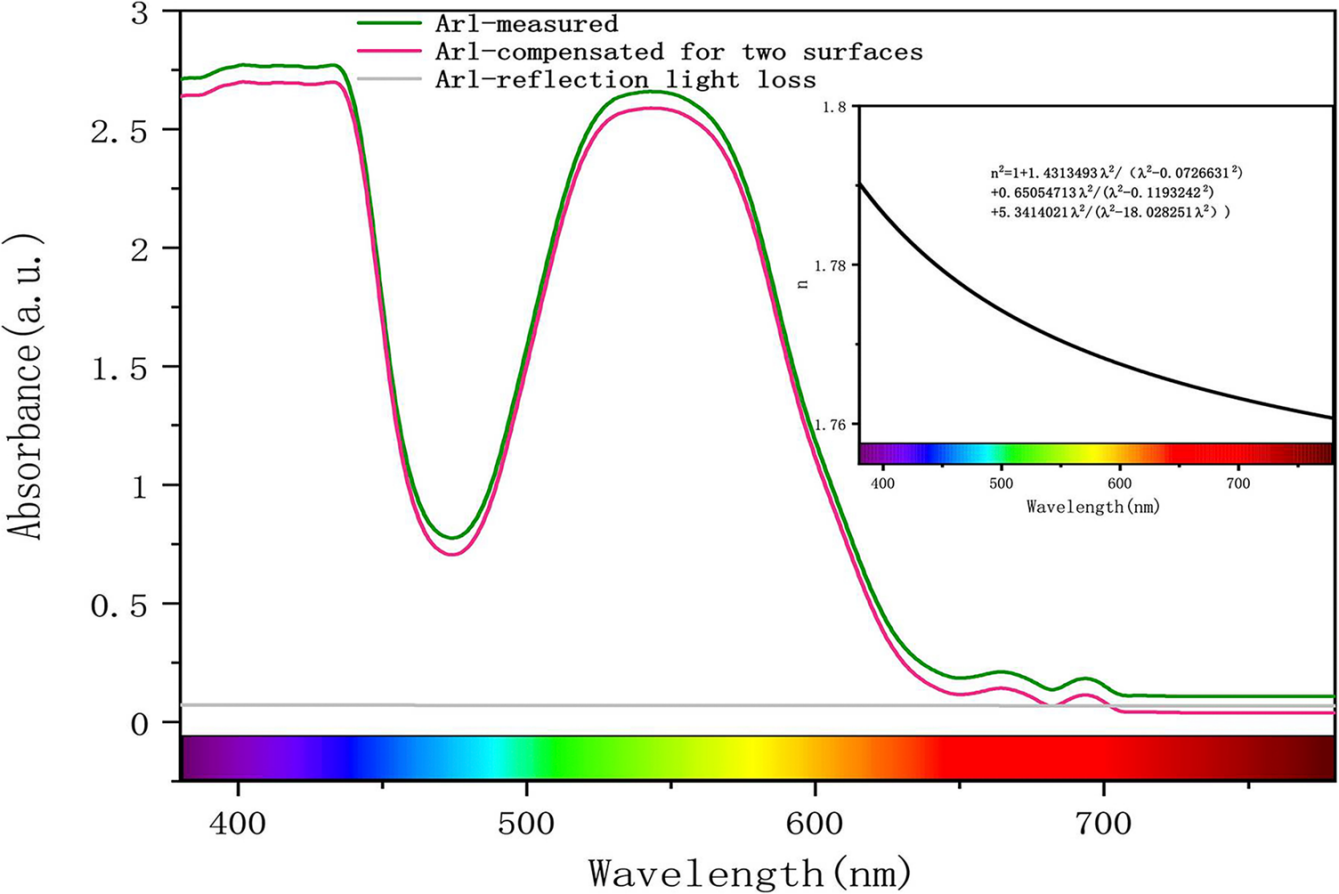
Corrected UV–Vis spectrum of the synthetic ruby R_oa_. The green line is the original spectrum, the pink line is the corrected spectrum, and the gray line is A_rl_. The top right is the curve of the relationship between the corresponding refractive index and wavelength.

Accurate visible spectroscopic measurements rely on correct calibration of the spectral baseline. Figure 3 shows the spectrum of baseline correction performed by the Sellmeier equation. The spectra of the synthetic ruby were converted to transmission spectra with light path lengths from 1 to 25 mm by multiplying the spectra by an appropriate value. That is I/I_0_, where "I" is the desired light path length and "I_0_" is the light path length of the reflection-corrected spectrum. By following the steps above, the ultraviolet–visible light spectrums corresponding to the light path length of 1 to 25 mm are obtained. The color matching function can be used to calculate the tristimulus value XYZ, and color space conversion can be used to obtain the color parameters L*, a*and b* in the CIE 1976 L*a*b* uniform color space^35,36^. The detailed conversion steps are detailed in the method. The synthetic ruby color parameters of the light path length from 1 to 10 mm are shown in Table 1.

**Table 1:** Synthetic ruby color parameters with light path length from 1 to 10 mm.

Light path length		R_oa_	R_ob_	R_ec_	Ru_oa_	Ru_ob_	Ru_ec_
1 mm	L* for daylight D65	81.72	81.86	83.79	80.7	78.07	83.71
	a* for daylight D65	25.72	25.14	12.85	24.49	22.82	9.58
	b* for daylight D65	− 5.4	− 2.41	− 3.62	− 1.94	− 2.09	3.02
	L* for incandescent light A	85.02	85.12	85.65	84.16	81.29	85.51
	a* for incandescent light A	29.91	29.36	15.95	28.98	27.63	12.82
	b* for incandescent light A	− 3.3	− 3.06	− 2.37	0.65	− 2.58	1.51
2 mm	L* for daylight D65	69.35	69.5	71.19	67.51	63.43	70.68
	a* for daylight D65	43.56	42.81	24.26	41.95	39.78	18.37
	b* for daylight D65	− 7.82	− 5.2	− 5.81	− 1.43	− 3.94	3.2
	L* for incandescent light A	74.79	74.9	74.72	73.28	68.94	74.09
	a* for incandescent light A	48.75	48.1	29.57	47.64	46.24	24.08
	b* for incandescent light A	− 3.39	− 3.09	− 3.08	3.79	− 1.38	3.6
3 mm	L* for daylight D65	60.77	60.89	61.50	58.37	53.66	60.38
	a* for daylight D65	54.55	53.88	34.14	53.03	50.88	26.37
	b* for daylight D65	− 7.62	− 5.34	− 6.65	1.02	− 2.77	3.95
	L* for incandescent light A	67.55	67.64	66.46	65.56	60.65	65.16
	a* for incandescent light A	59.25	58.73	40.64	58.16	56.9	33.67
	b* for incandescent light A	− 1.09	− 0.79	− 2.28	8.53	2.54	6.25
4 mm	L* for daylight D65	54.57	54.66	54.12	51.82	46.95	52.3
	a* for daylight D65	60.4	59.92	42.27	59.01	56.87	33.46
	b* for daylight D65	− 5.56	− 3.56	− 6.25	4.72	0.44	5.27
	L* for incandescent light A	62.16	62.23	60.23	59.83	54.79	58.22
	a* for incandescent light A	64.31	63.97	49.02	63.13	61.74	41.41
	b* for incandescent light A	2.64	2.89	− 0.2	13.97	7.88	9.42
5 mm	L* for daylight D65	49.87	49.93	48.49	46.91	42.12	46.00
	a* for daylight D65	62.93	62.63	48.52	61.50	59.18	39.42
	b* for daylight D65	− 2.44	− 0.66	− 4.78	8.94	4.69	7.12
	L* for incandescent light A	57.95	58.00	55.48	55.36	50.41	52.80
	a* for incandescent light A	66.21	66.01	54.82	64.80	63.05	47.25
	b* for incandescent light A	6.96	7.17	2.87	19.38	13.56	12.99
6 mm	L* for daylight D65	46.15	46.19	44.16	43.06	38.44	41.10
	a* for daylight D65	63.56	63.37	52.88	61.93	59.32	44.11
	b* for daylight D65	1.12	2.74	− 2.47	13.16	9.15	9.41
	L* for incandescent light A	54.52	54.57	51.78	51.72	46.91	48.53
	a* for incandescent light A	66.45	66.34	58.40	64.70	62.55	51.25
	b* for incandescent light A	11.37	11.56	6.54	24.35	18.91	16.79
7 mm	L* for daylight D65	43.10	43.13	40.78	39.92	35.48	37.25
	a* for daylight D65	63.16	63.06	55.56	61.24	58.34	47.45
	b* for daylight D65	4.74	6.25	0.40	17.07	13.36	12.02
	L* for incandescent light A	51.64	51.67	48.82	48.64	43.99	45.09
	a* for incandescent light A	65.84	65.79	60.27	63.75	61.23	53.67
	b* for incandescent light A	15.59	15.76	10.50	28.72	23.62	20.63
8 mm	L* for daylight D65	40.52	40.54	38.07	37.27	33.00	34.18
	a* for daylight D65	62.27	62.21	56.91	60.03	56.88	49.54
	b* for daylight D65	8.23	9.64	3.58	20.55	17.10	14.80
	L* for incandescent light A	49.14	49.17	46.37	45.98	41.47	42.25
	a* for incandescent light A	64.83	64.81	60.94	62.42	59.59	54.84
	b* for incandescent light A	19.49	19.65	14.47	32.46	27.64	24.32
9 mm	L* for daylight D65	38.28	38.31	35.84	34.98	30.86	31.68
	a* for daylight D65	61.13	61.11	57.30	58.58	55.24	50.57
	b* for daylight D65	11.48	12.82	6.82	23.56	20.32	17.59
	L* for incandescent light A	46.94	46.97	44.29	43.62	39.22	39.86
	a* for incandescent light A	63.64	63.64	60.83	60.93	57.86	55.10
	b* for incandescent light A	23.03	23.17	18.25	35.60	30.99	27.74
10 mm	L* for daylight D65	36.32	36.35	33.95	32.96	28.96	29.59
	a* for daylight D65	59.90	59.89	57.04	57.06	53.57	50.80
	b* for daylight D65	14.46	15.74	9.98	26.12	23.03	20.24
	L* for incandescent light A	44.96	44.99	42.47	41.50	37.19	37.78
	a* for incandescent light A	62.38	62.39	60.25	59.41	56.15	54.74
	b* for incandescent light A	26.19	26.33	21.76	38.21	33.73	30.78

### Color calculation and colorimetric parameter maps analysis

**Figure 4:**
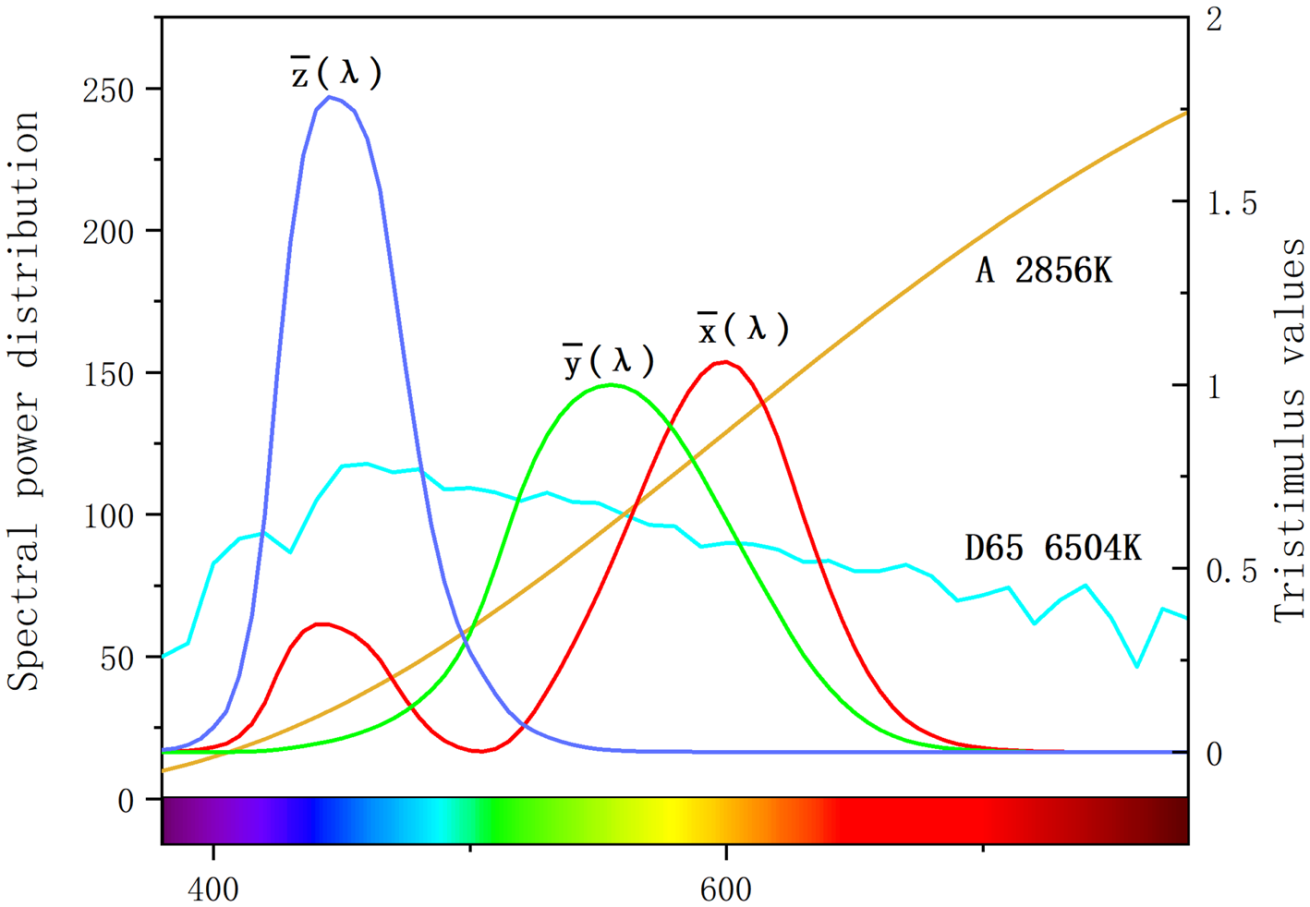
The spectral power distribution of the CIE D65 light source representing daylight (color temperature 6504 k), the spectral power distribution of the incandescent CIE A light source (color temperature 2856 k) and the color matching function spectrum ${\overline{\text{x}}}$(λ), ${\overline{\text{y}}}$(λ), ${\overline{\text{z}}}$(λ).

The color of transparent gems can be calculated based on the spectrum of the light source and the transmission spectrum of the gem. The integral of the spectral response curve corresponds to the signal emitted by the cone cells of the human eye. The reaction spectrum of the cone, the light source and the transmission spectrum of the sample are combined to determine the tristimulus value XYZ of the spectrum. The spectra of D65 light source and A light source used in this paper and the corresponding ${\overline{\text{x}}}$(λ), ${\overline{\text{y}}}$(λ), ${\overline{\text{z}}}$(λ) color matching function spectra are shown in Fig. 4. The light source is represented by a colorimeter through a standardized spectrum. CIE D65 light source represents the average daylight with a correlated color temperature of about 6504 k, and CIE A light source represents an incandescent lamp with a correlated color temperature of about 2856 k (D65:X = 95.04, Y = 100, Z = 108.87; A:X = 109.85, Y = 100, Z = 35.58).

The CIE XYZ color space is based on the uniform distribution of color perception, and colors will not be scattered in the Cartesian coordinate space. The calculated Euclidean distances between various color coordinates cannot be reasonably compared with each other. In the CIE 1976 L*a*b* color space, a* and b* represent horizontal and vertical Cartesian coordinate axes, L* represents the displacement perpendicular to the circular a*-b* grid. Different combinations of a* and b* can reproduce different tones, and the position of the color coordinate along L* indicates the brightness of the color. In the CIE 1976 L*a*b* color space, the tristimulus value XYZ is non-linearly converted into color parameters. And calculate the chromaticity value C* and hue angle h° under D65 light source and A light source. Taking sample R as an example, the absorption spectrum of light passing through synthetic ruby along any crystal direction can be calculated by the following formula:

11 $$\mathrm{Am}=x\times \text{R} \;  \text{down} \; \text{a}+y\times \text{R} \; \text{down} \; \text{b}+z\times \text{R} \; \text{ down} \; \text{c}$$

12 $${x}^{2}+{y}^{2}+{z}^{2}={r}^{2}$$

where Am is the absorption spectrum in either direction, R down a is Roa's UV–Vis absorption spectrum, R down b is Rob's UV–Vis absorption spectrum, R down c is Rec's UV–Vis absorption spectrum, x, y, z are the Cartesian coordinates of the ray path length, and the ray path length is constrained to lie on a sphere with a radius of r.

**Figure 5:**
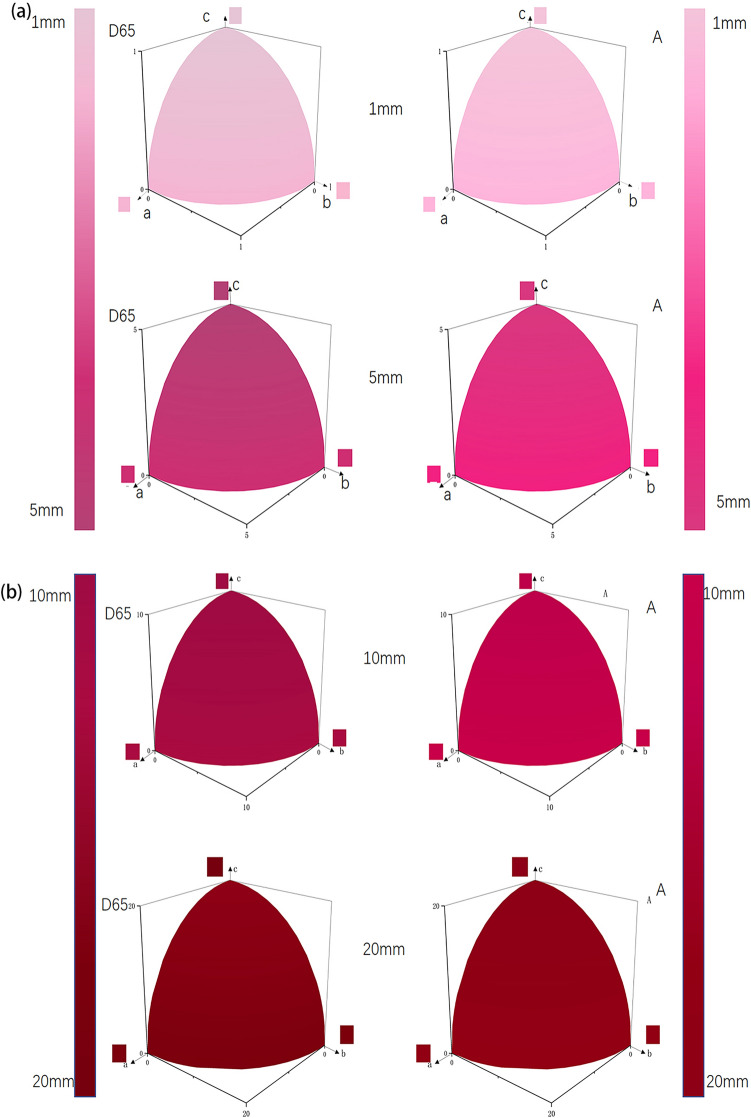
Synthetic ruby chromaticity diagram between R_oa_, R_ob_, and R_ec_ of different light path lengths. The left column is the D65 light source, and the right column is the A light source. (**a**) 1 mm and 5 mm chromaticity diagram, (**b**) 10 mm and 20 mm chromaticity diagram.

As shown in Fig. 5, the color of the synthetic ruby under D65 and A light source increases with the increase of the light path length, from the original light pink to purple-red and finally to deep red. When the light path length is 1 mm and 20 mm, the effect of different light sources on the color of ruby is not obvious. When the light path length is 5 mm and 10 mm, the color difference between the two light sources can be observed from the figure. Therefore, only when the length of the light path is in the appropriate range, that is, the length of the light path is not too large or too small, the light source will have a significant effect on the color of the ruby.

Figure 5 also shows the difference in colour between the a-axis (o-light direction) and the b-axis (e-light direction) of the ruby. This has to do with the pleochroism of ruby. When the light path length is 1 mm, the colour in the e light direction is light pink and the colour in the o light direction is pink; when the light path length is increased to 10 mm, the difference in colour between the e light direction and the o light direction becomes smaller; when the light path length is increased to 20 mm, the difference in colour between the e light direction and the o light direction is almost invisible by the naked eye. Therefore, it can be concluded that the light path length has an effect on the colour in the o light direction and the e light direction of the synthetic ruby. As the length of the optical path increases, the difference in colour between the o and e directions becomes smaller.

### The influence of light path length and light source on the color of synthetic ruby

**Figure 6:**
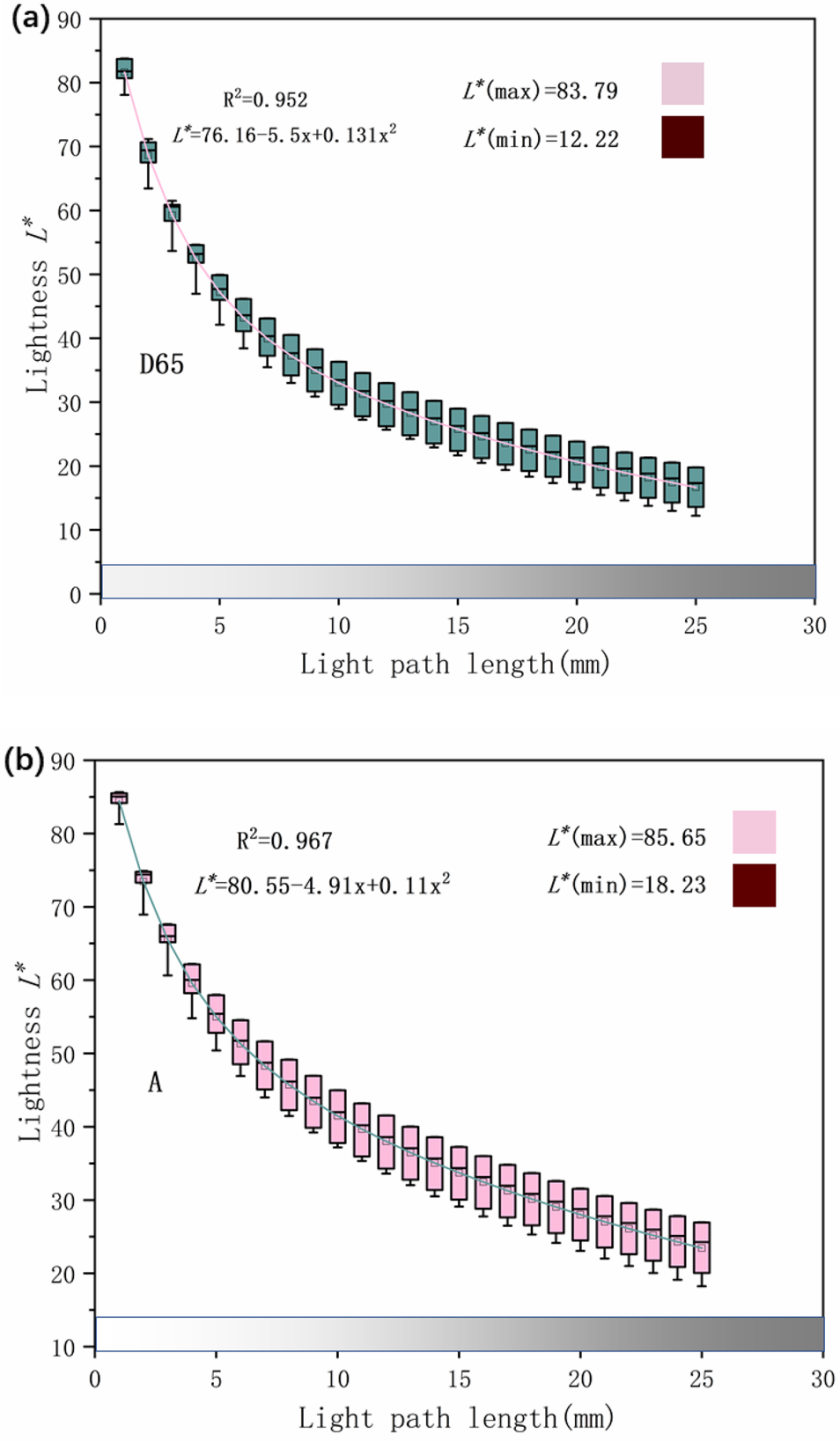
The lightness L* of synthetic rubies with different light path lengths under two light. (**a**) Synthetic ruby lightness L* under D65 light source. (**b**) Synthetic ruby lightness L* under A light source.

**Figure 7:**
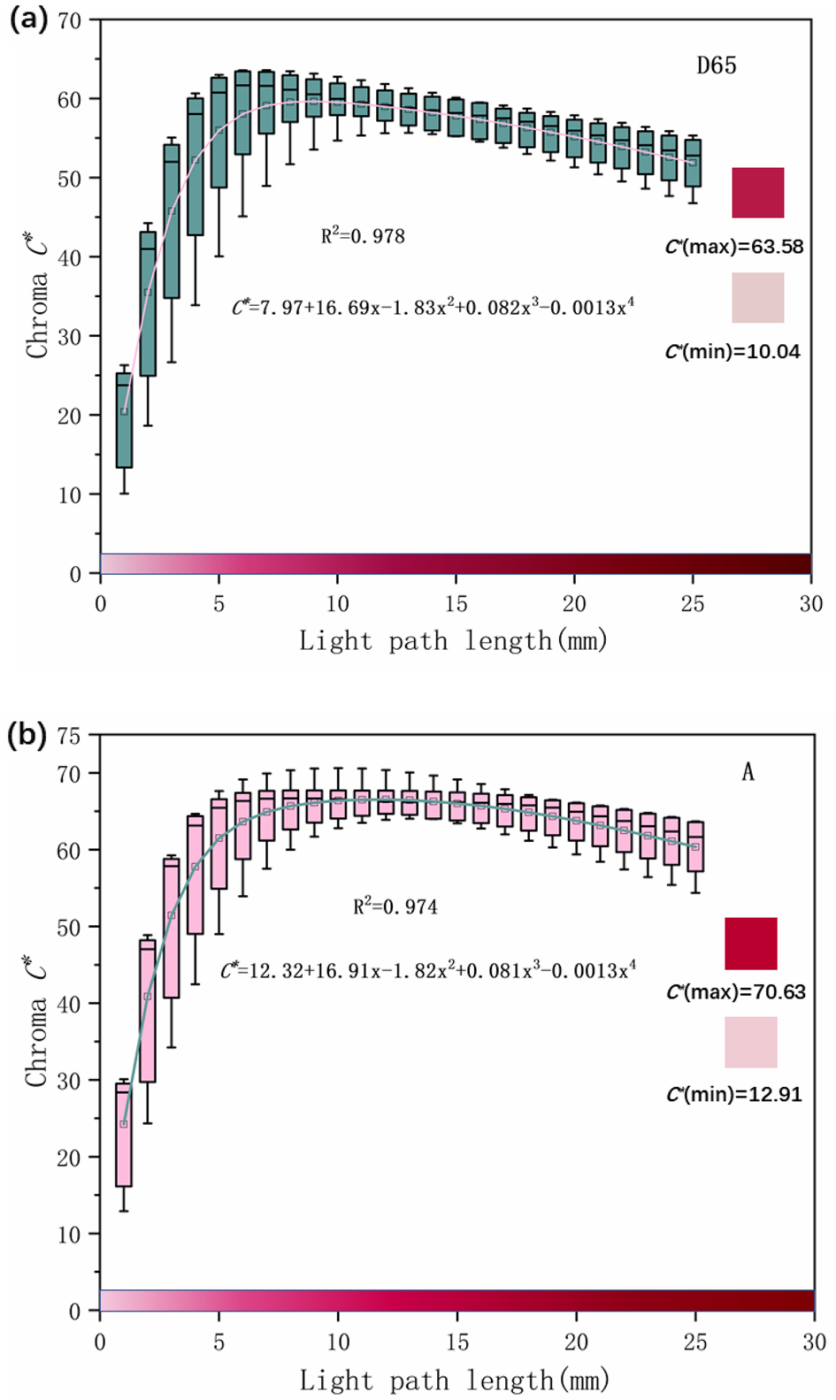
The chroma C* of synthetic rubies with different light path lengths under two light sources. (**a**) Synthetic ruby chroma C* under D65 light source. (**b**) Synthetic ruby chroma C* under A light source.

**Figure 8:**
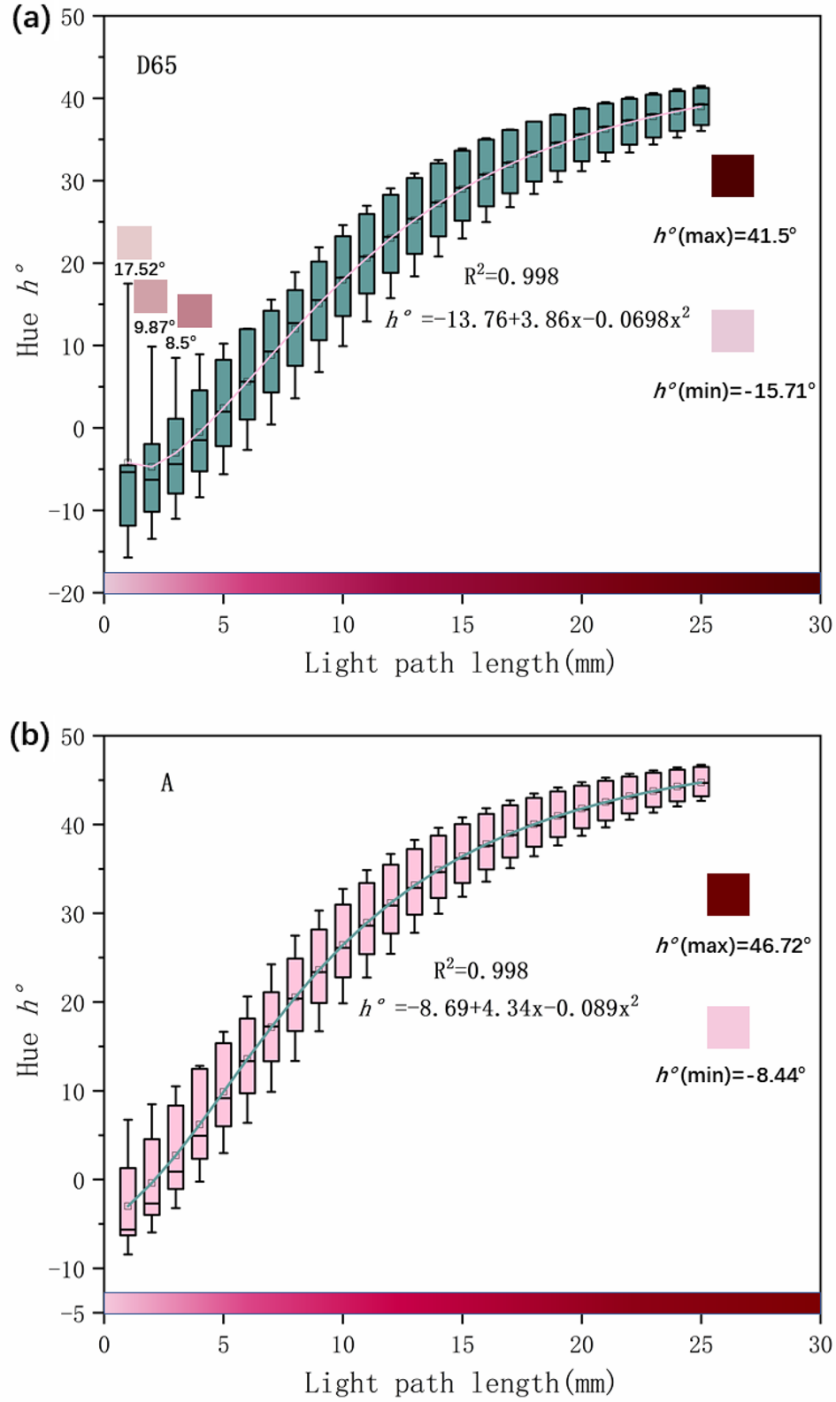
The hue angle of synthetic rubies with different path lengths under two light sources h° (**a**) D65 light source synthetic ruby hue angle h°. (**b**) A light source synthetic ruby hue angle h°.

Figures 6, 7, 8 show the sample color parameters L*, C*, h° for different light path lengths under D65 and A light sources. Take R_oa_, R_ob_, R_ec_, Ru_oa_, Ru_ob_, and Ru_ec_ with different light path lengths as a set of samples.

Figure 6 shows whether it is D65 light source or A light source, when the light path length increases, the lightness will decrease, showing a negative correlation (R^2^ = 0.952). This is consistent with the perception of the human eye. The lightness under the A light source is higher than the lightness under the D65 light source. When the length of the light path increases from 1 to 5 mm, the lightness drops rapidly, indicating that when the length of the light path is very small, its change has a significant impact on the lightness.

Figure 7 shows that under D65 and A light sources, the chroma C* of synthetic rubies with different light path lengths increases first and then decreases. Under the D65 light source, when the light path length is less than 7 mm, the chromaticity C* increases with the increase of the light path length, reaching the maximum value of 63.58; When the path length is greater than 7 mm, the color saturation reaches saturation and begins to decrease. Under A light source, it is bounded by 10 mm.

In addition, the chroma values of R_ec_ and Ru_ec_ under the two light sources are significantly lower than R_oa_ and Ru_oa_, which is caused by the pleochroism of the ruby. But under the D65 light source, when the light path length is 10 mm to 15 mm, the chroma value is close. This is because R_oa_ and Ru_oa_ reached the highest chroma and began to decline at 7 mm, while R_ec_ and Ru_ec_ continued to increase with the increase of the light path length because of the small chroma, and did not start to decrease until 12 mm. The color temperature of A light source is lower than that of D65 light source, so the chroma C* of R_oa_ and Ru_oa_ starts to decrease at 10 mm, while R_ec_ and Ru_ec_ start to decrease at 14 mm.

Figure 8 shows that the hue angle h° increases with the increase of the light path length under the two light sources, and the increasing amplitude gradually decreases. The hue angle under A light source is generally higher than that under D65 light source. But under the D65 light source, the hue angle h° decreases when Ru_ec_ (e light direction) is 1 mm to 3 mm, and h° begins to increase after 3 mm, this phenomenon does not appear under the A light source. This is because when the length of the light path increases, the color changes from pink to light pink, and the chromaticity coordinate a* increases significantly, resulting in a decrease in hue angle h°. The A light source has a yellow hue, making the hue angle h° greater than that under the D65 light source, so the hue angle will not decrease due to the increase in the length of the light path.

### The influence of light path length on the color difference $\Delta$E*_ab_

**Figure 9:**
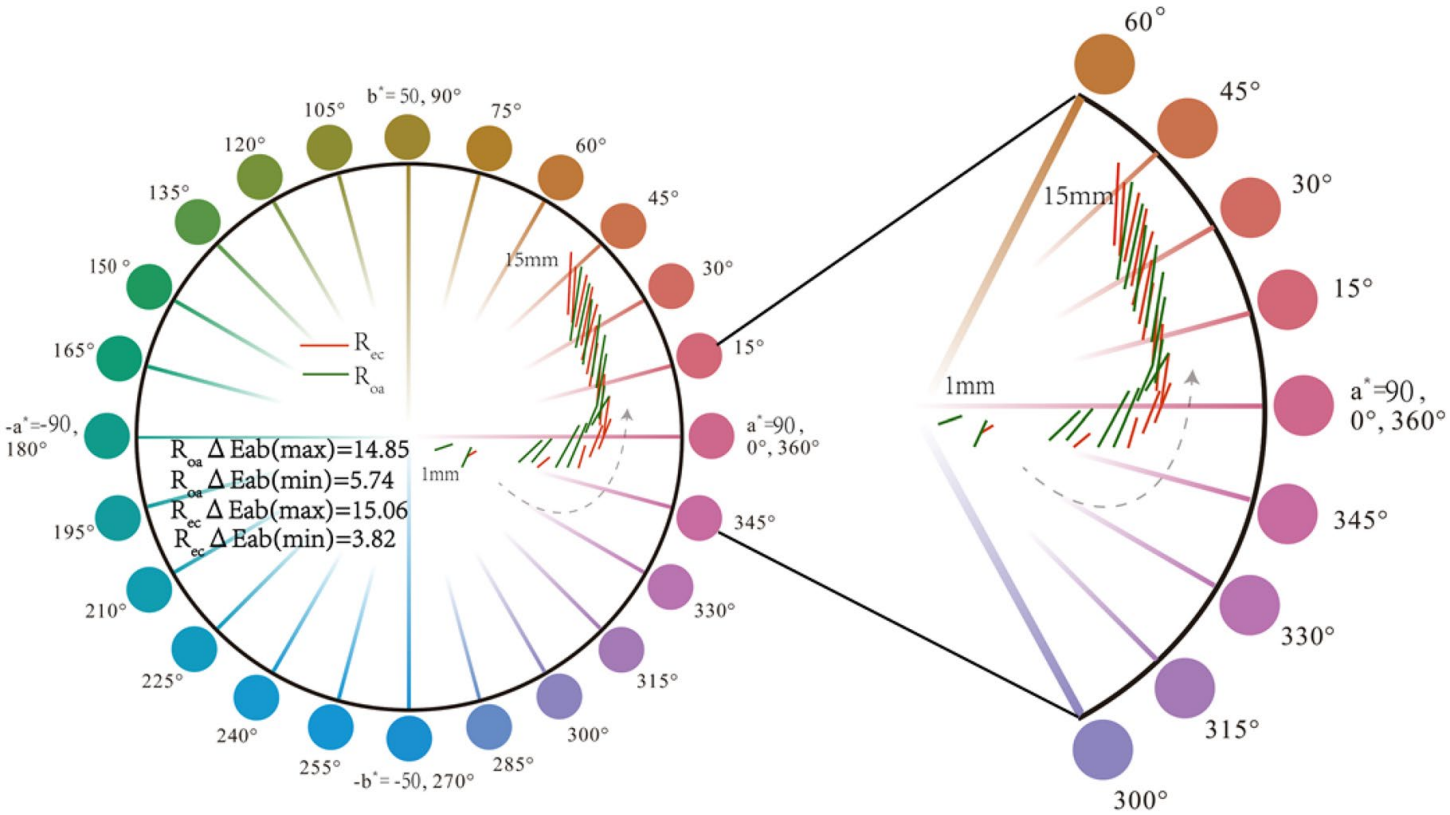
R_oa_ (o light direction) and R_ec_ (e light direction) color difference ΔE*_ab_ are distributed in two-dimensional a*b* plane, the left end of the connecting line is the color coordinate of D65 light source, and the right end is the color coordinate of light source A.

The color of synthetic ruby is plotted in CIE 1976 L*a*b* color space. In the three-dimensional space, the line connecting the points under the two light sources represents the Euclidean distance, which is the color difference ΔE*_ab_. Project the three-dimensional L*a*b* color coordinates to the two-dimensional a*b* plane (color circle), and draw a connecting line between the D65 light source and the A light source. In addition, link the change in the length of the light path with the color difference, draw a connecting line for each path length, and draw them in the same plane. The left end of the connecting line represents the color coordinate under the D65 light source, and the right end represents the color coordinate under the A light source. As shown in Fig. 9, take R_oa_ and R_ec_ as examples for drawing.

R_oa_ and R_ec_ are the o-light direction and e-light direction of synthetic ruby respectively. It can be seen from Fig. 9 that as the length of the light path increases, the connecting lines are distributed in an upward trend in the two-dimensional a*b* plane. The color difference $\Delta$E*_ab_ in R_oa_ and R_ec_ both increases first and then decreases. When the light path length is 12 mm, the color difference ΔE*_ab_ reach the maximum, which are 14.85 and 15.06 respectively. In order to further explore the influence of the light path length on the color when the light source changes, the light path length is increased to 25 m.

**Figure 10:**
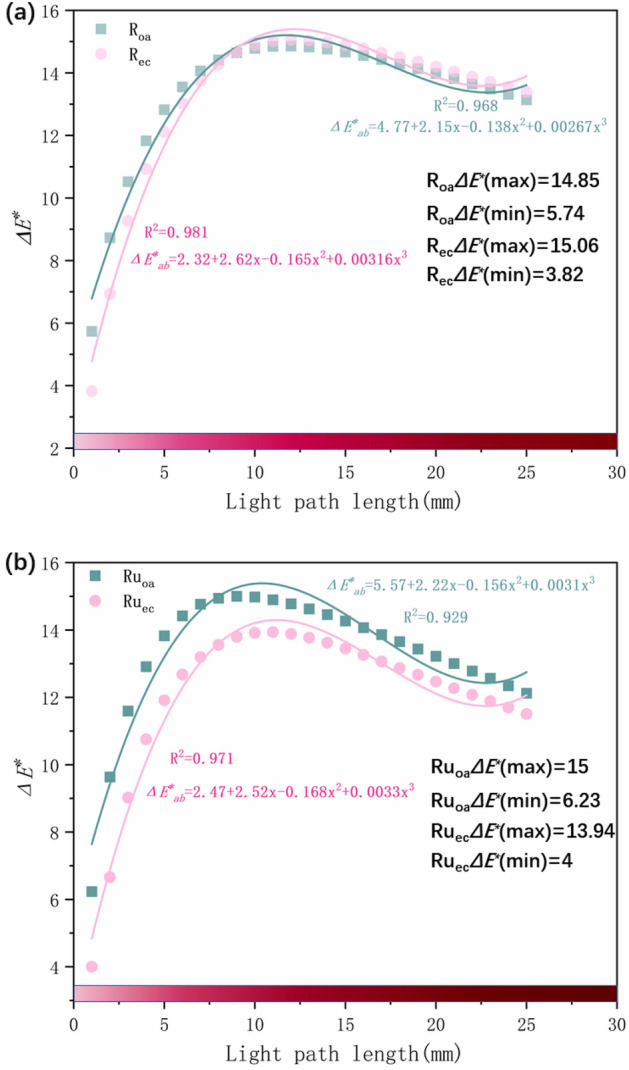
The color difference of different light path lengths in the o-light direction and e-light direction ΔE*_ab_ (**a**) R_oa_ and R_ec_ different light path length color difference $\Delta$E*_ab_, (**b**) Ru_oa_ and Ru_ec_ different light path length color difference ΔE*_ab_.

As shown in Fig. 10, increasing the light path length to 25 mm, the color difference $\Delta$E*_ab_ continues to decrease, indicating that as the light path length increases, the influence of the light source on the color is weakened. By comparing Fig. 10a,b, it is found that the color difference $\Delta$E*_ab_ changes of the two samples in the o-light and e-light directions are different, which is related to the color and pleochroism of the samples.

### The influence of UV–Vis absorbance peak area on the color of synthetic ruby

**Figure 11:**
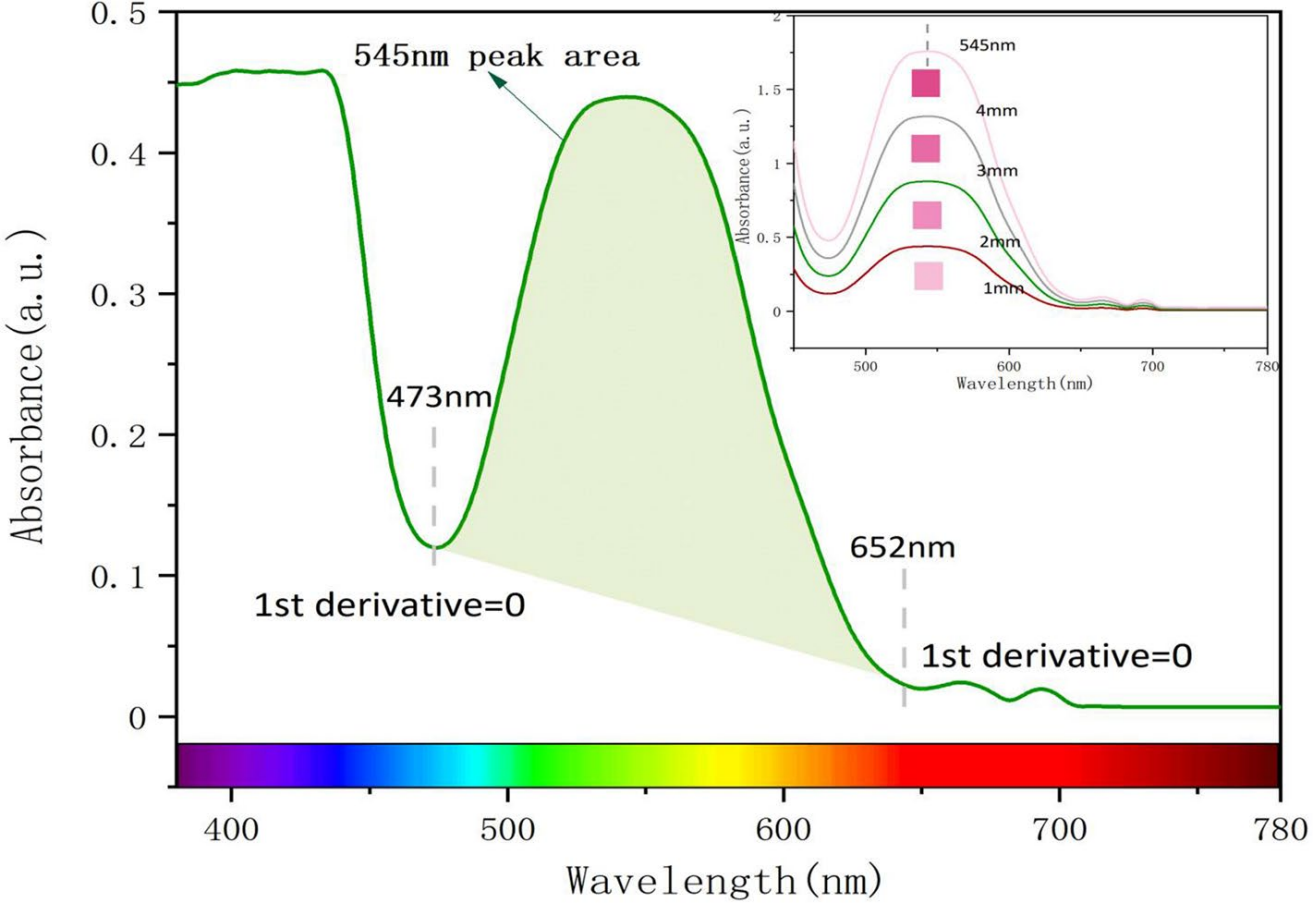
The absorption peak area (X) at 545 nm. Taking the sample R_oa_ as an example, the point where the first derivative is equal to zero is selected as the starting point and the end point of the 545 nm absorption peak range, and the absorption peak area is obtained by integrating from 473 to 652 nm. The top right is the ultraviolet–visible light absorption spectra of different light path lengths.

The strong absorption band at 545 nm in the ultraviolet–visible spectrum is caused by Cr^3+^, which absorbs yellow-green light in visible light and transmits red light, which has an important influence on the color of ruby. By calculating the first derivative of the ultraviolet–visible spectrum, the points with zero derivative near 473 nm and 652 nm are determined as the starting and ending points, and the absorption peak area at 545 nm is calculated (Fig. 11).

**Figure 12:**
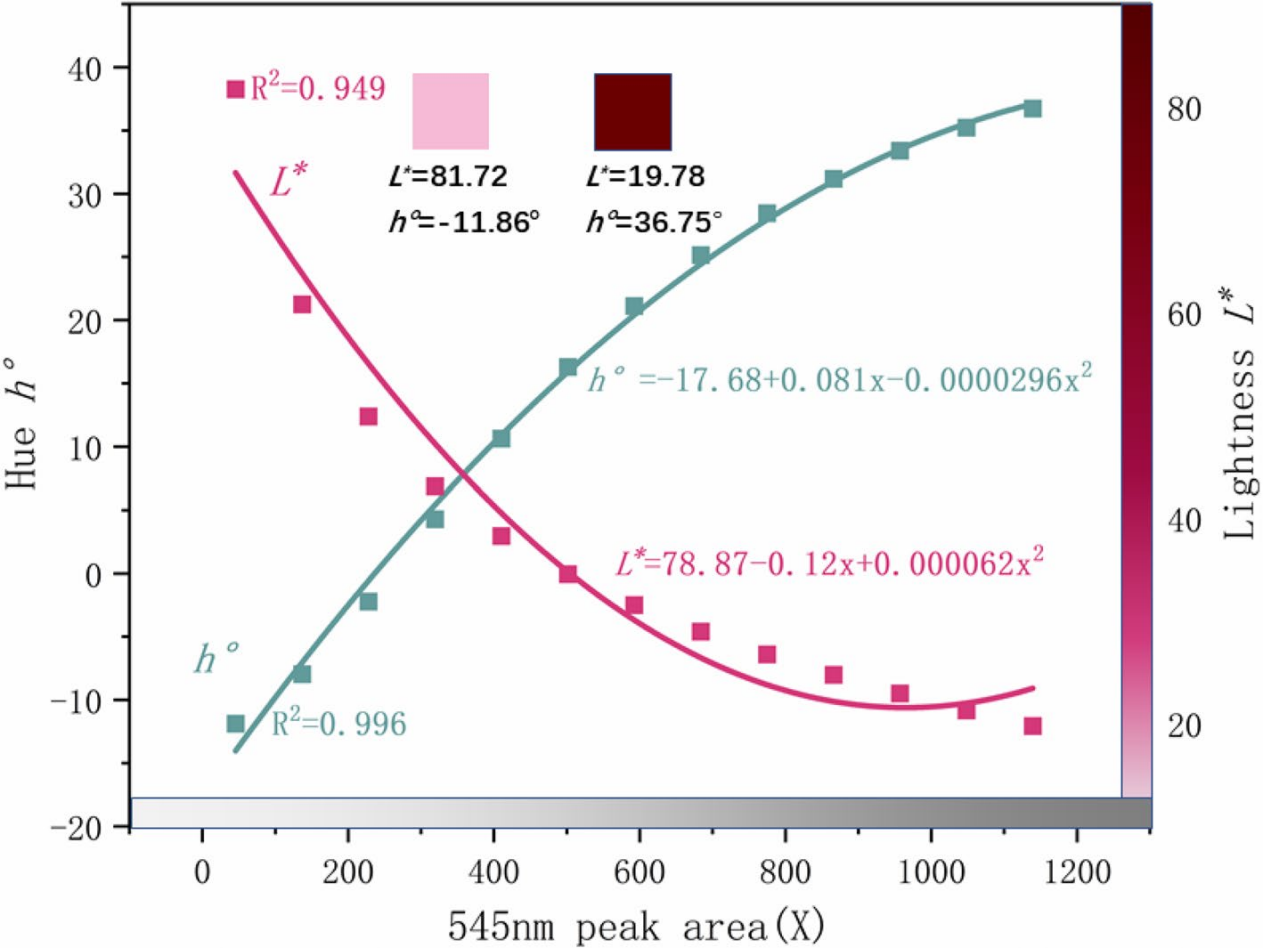
L* and h° of synthetic ruby with different UV–visible spectral peak areas under D65 light source.

Select a part of the data of different 545 nm absorption peak areas under D65 light source to plot 12. At the same time, it can be seen from Fig. 11 that the 545 nm absorption peak area is positively correlated with the light path length. Figure 12 shows that the hue angle h° is positively correlated with the peak area at 545 nm. When the absorption peak area increases, the hue angle h° increases. The R^2^ of the hue angle under the D65 light source is 0.996. h° changed from − 11.86 (that is, 348.14) to 36.75. The lightness L* is negatively correlated with the peak area at 545 nm. As the peak area increases, the ruby absorbs more visible light, and the lightness decreases. The chroma C* first increases and then decreases with the absorption peak area, which is related to the changes of L* and h°. When h° increases, L* decreases. When the length of the light path increases to a certain length, the decrease in brightness L* has a significant impact on chroma C*, making chroma C* begin to decrease.

## Conclusion

When the length of the light path is increased from 1 to 20 mm, the synthetic ruby changes colour from light pink to deep red. At the same time, due to the pleochroism of ruby, there is a difference in colour between the o light and e light directions of ruby. As the length of the light path increases, the difference becomes smaller.

Lightness L* increases as the length of the light path increases. When the light path length is 1 mm to 5 mm, its subtle changes will cause a significant decrease in lightness; The hue angle h° increases with the increase of the light path length, but under the D65 light source, when the light path length is 1 to 3 mm, the Ru_ec_ color shifts red, causing the hue angle h° to decrease; As the length of the light path increases, the chroma C* increases, but when the length of the light path increases to a certain length, the decrease of the lightness L* causes the chroma to start to decrease.

The color difference ΔE*_D65-A_ first increases and then decreases with the increase of the light path length. When the length of the light path reaches about 10 mm, the color difference reaches its maximum value. At this time, the light source has the greatest influence on the color. When the length of the light path continues to increase, the influence of the light source on the color is weakened.

In the UV–visible spectra, the strong absorption band at 545 nm caused by Cr^3+^ has a significant relationship with the colour of ruby. The strong absorption band at 545 nm has a positive correlation with the light path length. The larger the area of the band at 545 nm, the lower the lightness and the higher the hue angle, which means the ruby colour is redder.

## Methods

### UV–Vis spectroscopy

The UV-3600 UV–VIS spectrophotometer (Shimadzu, Tokyo, Japan) was used to carrry out the UV–Vis spectra. The test conditions were described as follows: the range of wavelength, 200–900 nm; slit width 2 nm; scanning speed medium; sampling interval 0.5 s; scanning mode, single.

### CIE1931 XYZ colour matching functions

The International Commission on Illumination proposed the CIEXYZ color system in 1931, and the tristimulus value XYZ can be obtained by matching the isoenergetic spectrum. The tristimulus value can calculate the color based on the spectrum collected from the surface of the object or the transmission through the object:

13 $$\mathrm{X}={\int }_{\uplambda }\mathrm{k\varphi }(\uplambda )\overline{\mathrm{x} }(\uplambda )d\lambda \approx \sum_{380}^{780}k\varphi (\lambda )\overline{x }(\lambda )\Delta\lambda $$

14 $$\mathrm{Y}={\int }_{\uplambda }\mathrm{k\varphi }(\uplambda )\overline{\mathrm{y} }(\uplambda )d\lambda \approx \sum_{380}^{780}k\varphi (\lambda )\overline{y }(\lambda )\Delta\lambda $$

15 $$\mathrm{Z}={\int }_{\uplambda }\mathrm{k\varphi }(\uplambda )\overline{\mathrm{z} }(\uplambda )d\lambda \approx \sum_{380}^{780}k\varphi (\lambda )\overline{z }(\lambda )\Delta\lambda $$

16 $$\mathrm{k}=\frac{100}{{\int }_{\uplambda }\mathrm{S}(\uplambda )\overline{\mathrm{y} }(\uplambda )\Delta\uplambda }$$

S($\uplambda$) is the relative spectral power distribution of the observation light source. For non-luminous objects, $\mathrm{\varphi }(\uplambda )$ is the product of the spectral transmittance $\mathrm{T}(\uplambda )$ and the relative spectral power of the light source S($\uplambda$), expressed as $\mathrm{T}(\uplambda )$ S($\uplambda$), or the product of the spectral reflectance $\mathrm{R}(\uplambda )$ and the relative spectral power distribution of the light source S($\uplambda$), expressed as $\mathrm{R}(\uplambda )$ S($\uplambda$). k is the naturalization coefficient. For non-luminous objects, the Y value of the selected standard illuminant was adjusted to 100^31,32^.

### Colour space conversion

In order to describe easily colors, the color tristimulus values in CIEXYZ are non-linearly converted to obtain the color parameters L*, a*, b* in the CIE1976L*a*b* color space system. The formula for conversion is as follows:

17 $${L}^{*}=116 \left( {\frac{X}{{{X_n}}}} \right)^\frac{1}{3}-16$$

18 $${a}^{*}=500\left[{ \left(\frac{X}{{X}_{n}} \right)}^\frac{1}{3}- \left( {\frac{Y}{{{Y_n}}}} \right)^\frac{1}{3}\right]$$

19 $${b}^{*}=200\left[{ \left(\frac{Y}{{Y}_{n}}\right)}^\frac{1}{3}- \left( {\frac{Z}{{{Z_n}}}} \right)^\frac{1}{3}\right]$$

For D65 light source, Xn = 95.04, Yn = 100, Zn = 108.88. For light source A, Xn = 109.85, Yn = 100, and Zn = 35.58. Xn, Yn, Zn are the colorimetric data obtained from the CIE1931 standard colorimetric observer (2°).

### CIE1976 L*a*b* colour system

The CIE1976L*a*b* color space is the most widely used in the field of colorimetry. The system consists of plane chromaticity axes a* and b* and vertical axis L*. a* stands for red, −a* stands for green; b* stands for yellow, −b* stands for blue. The chroma C* and hue angle h° can be calculated based on chromaticities a* and b*.

20 $${C}^{*}=\sqrt{{a}^{*2}+{b}^{*2}}$$

21 $$\mathrm{h}=\mathrm{arctan}\frac{{\mathrm{b}}^{*}}{{\mathrm{a}}^{*}}$$

To calculate the colour difference of rubies under different sources of illumination, we chose the CIE Lab (ΔE*_ab_) color difference formula:

22 $$\Delta {\mathrm{E}}_{\mathrm{ab}}^{*}=\sqrt{{(\Delta {L}^{*})}^{2}+({\Delta {a}^{*})}^{2}+({\Delta {b}^{*})}^{2}}$$

where $\Delta {\mathrm{a}}^{*}={\mathrm{a}}_{\mathrm{D}65}^{*}-{\mathrm{a}}_{\mathrm{A}}^{*}$, $\Delta \mathrm{b}={\mathrm{b}}_{\mathrm{D}65}^{*}-{\mathrm{b}}_{\mathrm{A}}^{*} \; \mathrm{ and } \;\Delta \mathrm{L}={\mathrm{L}}_{\mathrm{D}65}^{*}-{\mathrm{L}}_{\mathrm{A}}^{*}$, and $\Delta \mathrm{h}^\circ$ is the hue angle difference under different sources of illumination:

23 $$\Delta \mathrm{h}={\mathrm{h}}_{\mathrm{D}65}-{\mathrm{h}}_{\mathrm{A}}$$

where $\Delta {\mathrm{C}}^{*}$ is the chroma difference under different sources of illumination:

24 $$\Delta {\mathrm{C}}^{*}={\mathrm{C}}_{\mathrm{D}65}^{*}-{\mathrm{C}}_{\mathrm{A}}^{*}$$

## Data Availability

The dataset for this study is available from the corresponding author upon reasonable request.
